# Editorial: Metformin: Beyond Diabetes

**DOI:** 10.3389/fendo.2019.00851

**Published:** 2019-12-06

**Authors:** Frédéric Bost, Graham Rena, Benoit Viollet

**Affiliations:** ^1^Université Nice Côte d'Azur, Inserm U1065, Nice, France; ^2^University of Dundee, Dundee, United Kingdom; ^3^Université de Paris, Institut Cochin, CNRS UMR8104, INSERM U1016, Paris, France

**Keywords:** metformin, cancer, diabetes, neurodegenerative disease, mitochondria, gut microbiota, PCOS (polycystic ovarian syndrome)

Metformin, a member of the family of biguanides, is one of the most prescribed medications in the US and Europe and remains the first-line treatment for type 2 Diabetes (T2D) worldwide. It is a low cost medication, relatively well-tolerated, that has been given to millions of patients for more than 60 years in Europe. The literature on metformin is immense and recent discoveries in basic research place metformin on the short-list of the most promising drug for repurposing. Pioneering mechanistic studies demonstrating that metformin inhibits complex I in the respiratory chain of the mitochondria (1, 2) and the work of Zhou et al. showing that metformin activates AMP-activated protein kinase (AMPK) by inducing its phosphorylation at Thr^172^ (3), opened new horizons for maximizing clinical exploitation of metformin. Not only did they spur better understanding of metformin's action in T2D (4–6), but they also provided rational bases for laboratories to study the therapeutic potential of metformin outside of the conventional management of T2D. Twenty years on however, there still remains much debate regarding the key molecular target(s) of metformin. In this Research Topic, the evidence regarding direct effects of metformin on complex I of the electron transport chain and mitochondria are discussed in two focussed reviews (Fontaine; Vial et al.). They address topical research alongside earlier studies on the mechanism of action of metformin on mitochondrial complex I, how metformin modulates reactive oxygen species (ROS) production to prevent mitochondrial-mediated apoptosis and how the drug protects against permeability transition pore (PTP)-induced cell death. These effects are discussed in the context of T2D and cancer.

Metformin is now a well-established disruptor of cellular energy supply that targets the mitochondria [(7); Fontaine; Vial et al.]. The resulting compensatory changes on cellular metabolism to provide alternative sources of ATP and metabolites are detailed in this Research Topic by Andrzejewski et al.: including increased glycolysis, modifications of glutamine metabolism, and increase in PGC-1α [a major regulator of mitochondrial biogenesis also implicated in cancer (8)]. These adaptations are thought to play a central role in the resistance to metformin in cancer cells.

Activation of AMPK has been reported to inhibit the mechanistic target of rapamycin complex 1 (mTORC1) signaling pathway frequently activated in cancer cells (9). Furthermore, the tumor suppressor LKB1 was demonstrated to phosphorylate AMPK in response to biguanides (10). What then are the consequences in terms of cancer incidence in patients treated with metformin for decades? Observational evidence suggests that metformin reduces the incidence of cancer in people with diabetes (11). In this Research Topic three articles focus on the action of metformin on cancer and more specifically on melanoma (Jaune and Rocchi), leukemia (Biondani and Peyron), and colorectal cancer (Higurashi and Nakajima). These reviews describe in detail the recent advances concerning *in vivo* effects and the different molecular mechanisms underlying the anti-cancer action of metformin (AMPK dependent/independent effects, role of p53 and cellular effects: apoptosis, autophagy, proliferation, and cell migration), and present ongoing clinical trials for the prevention or treatment of various types of cancer.

One of the first reported benefits of metformin in reproductive biology was the increase of fertility in patients with polycystic ovary syndrome (PCOS) (12). This pathology is often associated with insulin resistance; thus, it is perhaps not surprising in hindsight that metformin ameliorates PCOS. Likewise, metformin has beneficial effects on obese male fertility (13). This important aspect of metformin action is addressed in a review that also discusses the potential epigenetic modifications induced by metformin in this context (Faure et al.). Among epigenetic modifications, histone acetylation/deacetylation plays a major role in the regulation of gene expression and metformin via AMPK was shown to regulate the expression of Sirtuin1 (Sirt1), a member of the class III (NAD+-dependent) histone deacetylases (HDACs) (14). An original research article of this collection by the group of J. Menendez, uses a computational approach to identify putative sites of interaction between Sirt1 and metformin (Cuyàs et al.). This is an important issue since metformin similarly to Sirtuins has been reported to expand longevity from yeast to mammals (15).

One of the most surprising effects of metformin found in recent years is its action on the gut microbiota. Indeed, the original discovery made by Oluf Pedersen's lab demonstrated that metformin causes a shift in the composition of microbiota altered during T2D (16). Two examples of the action of metformin on gut microbiota are given in two original research papers of the “Metformin: beyond diabetes” Research Topic (Wang et al.; Ji et al.).

Finally, there is growing evidence showing that metformin may have therapeutic potential in neurodegenerative disease. Rotermund et al. contribute a comprehensive review on the topic. In this article, evidence for effects of metformin on Alzheimer's disease, Parkinson's disease, amyotrophic lateral sclerosis, and Huntington's disease are summarized. Metformin has a protective action on neurons mainly because it protects from oxidative stress and neuroinflammation through mechanisms implicating mitochondria and glucose metabolism. Once again, cellular metabolism is at the forefront.

There are so many pathologies that have been shown to be impacted by metformin that it is a first-class candidate for drug repurposing in the near future. Besides cardiovascular disease (17, 18), tuberculosis (19) and very recently multiple sclerosis (20) may show promise. Future investigations and large-scale prospective clinical trials, some of them currently ongoing, will clarify this fascinating issue.

## Author Contributions

All authors listed have made a substantial, direct and intellectual contribution to the work, and approved it for publication.

### Conflict of Interest

The authors declare that the research was conducted in the absence of any commercial or financial relationships that could be construed as a potential conflict of interest.
